# Supplementary material for: Inflammation and cognition in severe mental illness: patterns of covariation and subgroups
Source: Mol Psychiatry. 2022 Dec 28;28(3):1284–92. doi: 10.1038/s41380-022-01924-w (PMC10005942; doi:10.1038/s41380-022-01924-w)
Supplement: Supplementary file 2 — Supplementary Tables [file 41380_2022_1924_MOESM2_ESM.pdf]

Supplementary tables

Supplementary table 1. Somatic medication use by SMI group

Somatic medications	SZ (N = 343)	BD (N = 289)
Anti-inflammatory/immunomodulatory, N (%)	11 (3.2)	1 (0.3)
Antidiabetics, N (%)	5 (1.5)	1 (0.3)
Cardiovascular/lipid modifying, N (%)	15 (4.4)	10 (3.5)
Antihistamines, N (%)	17 (4.9)	9 (3.1)
Gastrointestinal drugs, N (%)	13 (3.8)	7 (2.4)
*Other, N (%)	51 (14.9)	64 (22.1)

* Other includes vitamins, minerals, analgetics, thyroid agents, pulmonary agents, urological agents, musculoskeletal agents, contraceptives, sex hormones, anxiolytics, anti-inflammatory (local administrative agents, i.e. crème/inhalators), hematological agents, parenteral nutrition agents, substance dependency agents and mucolytic agents.
Abbreviations: Schizophrenia (SZ), Bipolar disorder (BD), Healthy controls (HC)

Supplementary Table 2. Overview of cognitive domains and corresponding tests

***Domain*/Test**	**SZ**<br>Battery 1 (N=238)<br>Battery 2 (N=105)	**BD**<br>Battery 1 (N=167)<br>Battery 2 (N=122)	**HC**<br>Battery 1 (N=255)<br>Battery 2 (N=515)	**Test battery**
Fine-motor speed				
Grooved Pegboard Test (Halstead-Reitan)	-0.4 (1.2)	-0.1 (1.0)	0.3 (0.6)	1 & 2
Psychomotor processing speed				
Symbol Coding (WAIS-III)	-0.5 (1.0)	0.0 (1.0)	0.5 (0.8)	1
BACS Symbol Coding (MCCB)	-0.7 (1.0)	-0.1 (1.0)	0.4 (0.8)	2
Mental processing speed				
Color naming & Color reading (D-KEFS)	-0.4 (1.0)	-0.0 (1.0)	0.2 (0.8)	1 & 2
Attention				
Digit span forward (WAIS-III)	-0.3 (1.0)	-0.2 (1.0)	0.1 (0.9)	1 & 2
Verbal learning				
Total recall (CVLT-II)	-0.4 (1.0)	0.1 (1.0)	0.3 (0.8)	1
Total recall (HVLT-R, MCCB)	-0.5 (1.1)	0.1 (1.0)	0.3 (0.8)	2
Verbal memory				
Long-delay free recall (CVLT-II)	-0.4 (1.0)	0.1 (1.0)	0.3 (0.8)	1
Delayed recall (HVLT-R, MCCB)	-0.6 (1.2)	0.1 (0.8)	0.2 (0.7)	2
Semantic fluency				
Category fluency (D-KEFS)	-0.6 (1.0)	0.1 (1.0)	0.5 (0.7)	1
Category fluency (MCCB)	-0.7 (1.0)	0.0 (1.0)	0.3 (0.9)	2
Working memory				
Letter Number Sequencing (WAIS-III)	-0.4 (0.9)	-0.1 (1.0)	0.4 (0.9)	1
Letter Number Sequencing (MCCB)	-0.4 (1.0)	-0.1 (0.9)	0.2 (0.9)	2
Cognitive control				
Inhibition/inhibition switching (D-KEFS)	-0.4 (1.1)	-0.1 (1.0)	0.2 (0.7)	1 & 2

[a]Mean (standard deviation, SD) of Z-scores per test
Note: To increase N because two different test batteries have been used, we standardized the tests separately before collapsing. Domains are named based on the function the tests measure, although we acknowledge that some tasks tap into multiple functions and other domain names could have been chosen.
Abbreviations: Schizophrenia (SZ), Bipolar disorder (BD), Healthy controls (HC)

Supplementary table 3. Stability of markers stored for 24 hours at 4°

	BL	2h	4h	24h	Avg CV%
BAFF	100	120	97	113	10,2
APRIL	100	114	104	94	8,0
S100B	100	107	105	106	3,0
Furin	100	129	99	98	14,1
GFAP	100	104	97	90	6,1
ENO2	100	113	103	86	11,2
A2M	100	90	80	93	9,0
GROα	100	84	99	89	8,3
SDF1α	100	101	90	98	4,9
Eotaxin	100	113	100	92	8,8
RANTES	100	76	92	82	12,1
MADCAM	100	93	104	104	5,1
JAMA	100	100	132	123	14,5
NCAD	100	108	99	99	4,2
ICAM1	100	92	94	100	4,2
VCAM1	100	85	94	102	8,0
PSEL	100	82	102	95	9,3
SA3	100	112	98	106	6,1
IL18	100	100	103	104	1,8
IL18BPa	100	94	101	99	3,0
IL18RAP	100	96	95	110	7,0
HNP1-3	100	100	105	107	3,5
BD1	100	79	94	98	10,2
BD2	100	89	132	124	17,8

Supplementary table 4. Percentage missing per inflammatory/immune marker

Inflammatory/immune markers	SZ (N = 343)	BD (N = 289)	HC (N = 770)	Total (N = 1402)
			Percentage (%) missing	
BAFF	10.2	10.3	9.6	9.9
APRIL	4.6	6.9	5.4	5.5
S100B	12.2	8.3	9.8	10.1
Furin	7.8	5.1	6.6	6.6
GFAP	12.2	10.7	12.3	11.9
ENO2	3.2	4.4	7.5	5.8
A2M	4	1.3	3.1	2.9
GROα	4.6	5.5	5.8	5.4
SDF1α	7.2	5.5	8.8	7.7
Eotaxin	2.6	4.1	4.6	4
RANTES	2.6	4.8	3.7	3.7
MADCAM	4.3	2.4	2	2.7
JAMA	3.2	3.4	5.9	4.7
NCAD	12.8	9.3	8.5	9.7
ICAM-1	6.9	3.4	1.6	3.3
VCAM-1	2.6	3.1	2.9	2.9
PSEL	2.9	4.1	3.3	3.4
SA3	4.6	3.1	3.5	3.7
IL-18	9.3	10	7	8.2
IL-18BP	9.6	5.5	2.5	4.9
IL-18RAP	12.2	11.7	13.7	12.9
IL-18R1	5.8	4.8	3.8	4.5
HNP13	11	12.1	14.1	12.9
BD-1	4.6	6.5	5.7	5.6
BD-2	11.3	8.9	6.1	7.9

Note: Missing data was imputed using Multiple Imputation by Chained Equations (MICE). See Supplementary figure 1 (A-C) for density distributions of observed and imputed data.
Abbreviations: Schizophrenia (SZ), Bipolar disorder (BD), Healthy controls (HC)

Supplementary Table 5. Inflammatory/immune-related marker levels between SZ, BD and HC

Immune/Inflammatory markers[a]	SZ (N = 343)	BD (N = 289)	HC (N = 770)	*p*-value[b]	Effect size[c]	Pairwise comparisons[b]
Neuroinflammation						
A2M (µg/mL)	14.6 (9.3)	14.3 (10.0)	13.5 (8.6)	**0.04**	0.003	ns
APRIL (pg/mL)	235.5 (196)	249.7 (204)	326 (237)	**<0.001**	0.06	BD, SZ<HC
BAFF (pg/mL)	238 (123)	215 (119)	226 (112)	**0.04**	0.003	ns
SA3 (µg/mL)	1.2 (0.8)	1.3 (0.8)	1.2 (0.8)	**0.01**	0.005	HC, SZ<BD
BBB integrity						
ENO2 (ng/mL)	1.8 (3.1)	2.6 (3.4)	3 (4.7)	**<0.001**	0.03	SZ<BD, HC; BD<HC
Furin (ng/mL)	0.4 (0.2)	0.3 (0.2)	0.4 (0.2)	**0.03**	0.003	BD<SZ, HC
GFAP (pg/mL)	139 (78.5)	139 (100)	139 (107)	ns	-	-
S100B (ng/mL)	104 (26.1)	101.2 (21.8)	104 (26.1)	**<0.01**	0.006	BD<SZ, HC
Chemokines						
Eotaxin (pg/mL)	122.4 (74.9)	131 (66.1)	138.5 (81.6)	**<0.001**	0.02	SZ, BD<HC
GROα (pg/mL)	19.5 (13)	20.4 (14.7)	21.7 (14.9)	**<0.01**	0.006	SZ<HC
RANTES (ng/mL)	77 (56.3)	74.2 (73.3)	90.3 (76)	**<0.001**	0.01	SZ, BD<HC
SDF1α (pg/mL)	1437 (716)	1497 (816)	1577 (799)	**<0.01**	0.008	SZ<HC
Cell adhesion molecules						
ICAM-1 (ng/mL)	260.3 (95)	268.6 (99)	249.7 (94)	**<0.01**	0.006	HC<BD
JAMA (ng/mL)	1.2 (0.7)	1.2 (0.7)	1.1 (0.7)	**0.02**	0.004	HC<BD,SZ
MadCAM-1 (ng/mL)	7.5 (3.4)	7.3 (4.1)	6.8 (3.6)	**<0.01**	0.007	HC<SZ
NCAD (ng/mL)	6.4 (2.4)	6.7 (2.2)	6.6 (2.4)	ns	-	-
PSEL (ng/mL)	39.9 (27.4)	41.3 (31)	45.2 (31.5)	**<0.01**	0.005	SZ<HC
VCAM-1 (ng/mL)	467 (143)	480 (156)	497 (146)	**<0.001**	0.009	SZ<HC
IL-18 system						
IL-18 (pg/mL)	795 (1238)	694 (1016)	691 (1031)	ns	-	-
IL-18BP (ng/mL)	6.2 (2.6)	5.8 (2.2)	5.4 (2.2)	**<0.001**	0.02	HC<BD, SZ
IL-18RAP (pg/mL)	45.5 (12.3)	45.4 (12.7)	46.8 (13.7)	ns	-	-
IL-18R1 (ng/mL)	0.81 (0.4)	0.8 (0.4)	0.7 (0.4)	**0.03**	0.003	HC<SZ
Defensins						
HNP1-3 (ng/mL)	7 (3.2)	6.9 (2.6)	7.3 (4)	**<0.01**	0.007	BD, SZ<HC
BD-1 (ng/mL)	11.3 (4.5)	12.7 (5.4)	11.9 (4.5)	**<0.001**	0.01	SZ<BD, HC; HC<BD
BD-2 (ng/mL)	254 (211)	229(207)	199 (182)	**<0.001**	0.01	HC<SZ, BD

[a]Median (interquartile range, IQR)

[b]Kruskal-Wallis test and Dunn's pairwise comparison (Bonferroni corrected)

[c]eta-squared based on H statistic: 0.01 -< 0.06 (small), 0.06 -< 0.14 (moderate), >=0.14 (large)

Abbreviations: Schizophrenia (SZ), Bipolar disorder (BD), Healthy controls (HC)

Supplementary Table 6. Cognitive domain scores between SZ, BD and HC

**Cognitive domains**[a]	**SZ (N = 343)**	**BD (N = 289)**	**HC (N = 770)**	***p*-value**[b]	**Effect size**[c]	**Pairwise comparisons**[b]
Fine motor speed	-0.42 (1.18)	-0.14 (1.01)	0.31 (0.56)	**<0.001**	0.43	SZ<HC,BD;BD<HC
Psychomotor processing speed	-0.57 (0.94)	-0.02 (0.95)	0.41 (0.83)	**<0.001**	0.49	SZ<HC,BD;BD<HC
Mental processing speed	-0.42 (1.00)	-0.05 (0.95)	0.26 (0.73)	**<0.001**	0.41	SZ<HC,BD;BD<HC
Attention	-0.31 (0.87)	-0.16 (0.89)	0.12 (0.94)	**<0.001**	0.24	SZ,BD<HC
Verbal learning	-0.41 (1.04)	0.14 (0.99)	0.27 (0.84)	**<0.001**	0.39	SZ<BD,HC
Verbal memory	-0.44 (1.09)	0.12 (0.92)	0.22 (0.85)	**<0.001**	0.36	SZ<HC,BD;BD<HC
Semantic fluency	-0.63 (0.86)	0.06 (0.98)	0.37 (0.86)	**<0.001**	0.54	SZ<HC,BD;BD<HC
Working memory	-0.42 (0.92)	-0.13 (0.90)	0.33 (0.96)	**<0.001**	0.41	SZ<HC,BD;BD<HC
Cognitive control	-0.39 (1.09)	-0.09 (1.01)	0.26 (0.65)	**<0.001**	0.35	SZ<HC,BD;BD<HC

[a]Mean (standard deviation, SD), Z-scores

[b]Robust one-way ANOVA and lincon post-hoc for pairwise comparison (multiple comparison corrected; WRS2 R-package)[1]

[c]Explanatory measure of effect size, ξ (0.1, 0.3, 0.5, small/medium/large; WRS2 R-package)

Abbreviations: Schizophrenia (SZ), Bipolar disorder (BD), Healthy controls (HC)

[1] Patrick Mair and Rand Wilcox, ‘Robust Statistical Methods in R Using the WRS2 Package’, *Behavior Research Methods*, 52.2 (2020), 464–88 (p. 2) <https://doi.org/10.3758/s13428-019-01246-w>.
